# Transcriptome-Based Analysis of Phosphite-Induced Resistance against Pathogens in Rice

**DOI:** 10.3390/plants9101334

**Published:** 2020-10-09

**Authors:** Yuqing Huang, Shengguan Cai, Guoping Zhang, Songlin Ruan

**Affiliations:** 1 Institute of Crop Science, College of Agriculture and Biotechnology, Zhejiang University, Hangzhou 310000, China; caisg@zju.edu.cn (S.C.); zhanggp@zju.edu.cn (G.Z.); 2 Institute of Crop Science, Hangzhou Academy of Agricultural Sciences, Hangzhou 310000, China

**Keywords:** phosphite, gene expression, pathogen resistance

## Abstract

Phosphite (PHI) has been used in the management of *Phytophthora* diseases since the 1970s.We assessed the effect of PHI on controlling the incidence of *Xanthomonas oryzae pv.oryzae* and *Pyricularia grisea*. As a result, PHI application significantly inhibited the incidence of the diseases. To clarify the molecular mechanism underlying this, a transcriptome study was employed. In total, 2064 differentially expressed genes (DEGs) were identified between control and PHI treatment. The key DEGs could be classified into phenylpropanoid biosynthesis (ko00940), starch and sucrose metabolism (ko00500), and plant hormone signal transduction (ko04075). The expressions of defense-related genes had a higher expression lever upon PHI treatment. This study provides new insights into the mechanism of protection effect of PHI against pathogens.

## 1. Introduction

Phosphorus (P) is among the critical macronutrients required by all living organisms. It is essential for plant growth and development; thus, the deficiency of P severely limits the growth and the yield in crops [1]. In the agricultural system, element P tends to form compounds combined with other elements, such as oxygen (O) or hydrogen (H). When fully oxidized, it exists as a phosphate molecule. However, it forms a phosphite (PHI) when not fully oxidized. The solubility, uptake efficiency, and the effect on plant metabolism vary between phosphate and PHI due to the simple change in molecular form [2]. Phosphate compounds are the most utilized form that could be supplied to plants as P fertilizer. It takes more than 6 months for PHI to be oxidized to phosphate [3]. The solubility of PHI is high, so it is more easily obtained by plants. When plants are supplied with PHI as the sole P source, the growth of plants is severely restrained, root hairs form, and the expression of high-affinity transporters genes are inhibited [4]. The phytotoxicity of PHI depends on the phosphate status in plant. The toxicity of PHI will be obvious if phosphate is insufficient. In higher concentrations of PHI, a dramatic reduction in shoot and root mass of rice was observed. The root hair elongation was slightly affected due to the increasing concentration of PHI [5].

Pathogens are the main threats to the sustainability and cause severe damage to agricultural industry. PHI could induce stimulation of defense responses to plants if applied at appropriate concentrations. Some chemicals such as PHI at lower concentrations have been used extensively in the management of Phytophthora diseases. Many fertilizers containing PHI are available on the market. The effect of PHI in controlling diseases caused by oomycetes was discovered in 1977 [6].It is widely used due to its low cost and long-lasting defense effect against diseases. PHI is currently utilized as fungicides primarily rather than fertilizers in field crops. Cucumber and tomato plants which are pretreated with PHI react more rapidly to pathogen attack, mainly benefiting from the accumulation of phenolic-like compounds [7]. A similar finding was revealed by Araujo et al.: the concentrations of two alkaloids and 10 phenolic compounds were higher in the PHI-treated tissues than the control [8]. The application of PHI as fungicides worked better preventatively than curatively. Foliar applications of PHI on post-harvest potato tubers reduced their susceptibility to specific diseases caused by oomycete pathogens such as *Phytophthora infestans* [9].

The increase in peroxidase and polyphenol oxidase activities contributed to the defense mechanism of PHI applications [9]. The protection pattern of PHI application mainly includes the inhibition of pathogens growth directly, accumulation of stress-related metabolism and the expression of defense genes [10]. The protection effect of PHI application on pathogens acts in a dose-dependent manner. Low concentration of PHI induces the synthesis of defense genes, phytoalexins, and phenolic compounds. While under high concentrations, PHI could inhibit the growth of pathogens directly [10,11]. Tomato and pepper plants showed typical symptoms of phosphorus deficiency when supplied with PHI as sole source of nutrient, but their susceptibility to crown rot was much lower than phosphate-treated plants [12].

PHI induces the resistance against pathogen attack via priming. PHI is used as a compound which could induce a systemic defense response to pathogens. The mechanism by which it operates remains to be elucidated. Systemic acquired resistance (SAR) is one type of induced resistance (IR) which induces the protection of plants from some pathogens [13]. It is associated with salicylic acid (SA) and pathogenesis-related proteins (PRs). Induced systemic resistance is another type of IR, which needs the signal molecular jasmonate (JA) and ethylene (ET) [14]. Priming is a process in which plants acquire enhanced resistance to pathogens with the function of some plant hormones and other chemical compounds [15]. The phenomenon prime defenses for enhanced response to pathogens is activated by the treatment of plants with some natural or synthetic compounds, such as SA, β-aminobutyric acid (BABA) or PHI [16]. ROS production is boosted, and callose and pathogenesis-related genes are accumulated in priming responses. However, the mechanisms underlying priming remains poorly understood. Under low PHI concentrations, SA and pathogenesis-related protein1 were accumulated, increasing resistance against pathogen attack in the future [17]. 

The transcription of defense genes *PR1*, *PR5*, *THI2.1*, and *PDF1.2* was enhanced after the application of PHI in Ler accession. These genes were involved in salicylic acid and jasmonic acid/ethylene pathways. The response to infection was primed rapidly which occupied the activation of a set of defense genes relating to SA and JA/ET pathways [16]. 

In the present study, we analyzed the role of PHI in controlling *Xanthomonas oryzae pv.oryzae* and *Pyricularia grisea*. To gain better insight into the mechanism of PHI-mediated plant defense against pathogens, Illumina RNA-sequencing technology was applied to explore the global transcriptome of PHI-treated rice seedlings. The results obtained will contribute to understanding the mechanism of priming gained after PHI treatment.

## 2. Materials and Methods

### 2.1. Sample Preparation

Rice seeds of Nipponbare (*O. sativa ssp. japonica*) were soaked in distilled water for 24 h at 30 °C and transferred to 150 ml agar media containing 50 ppm PHI. Seedlings were kept in a growth chamber under 25 °C and controlled humidity conditions. Leaves of five seedlings were collected for RNA-sequencing for each replicate two weeks after germination. There were three replicates for each sample. Leaf images of section slides were taken using an optical microscope. The parameters of leaf thickness were analyzed with Image J software (NIH, USA) and calculated with Excel.

### 2.2. Disease Assessment

The *Xanthomonas oryzae pv.oryzae* and *Pyricularia grisea* inoculations were conducted on two-week-old rice seedlings of control and 50 ppm PHI application, and the incidence of infection was calculated after two weeks. Two hundred seeds of each replicate were used for each replicate. Three replicates were performed for each sample. Spray leaf assay was used for disease assessment. The spores of *Pyricularia grisea* (3 × 10^5^ spores/ml) and *Xanthomonas oryzae pv.oryzae* (3 ×10^5^ spores/ml) was sprayed on the leaves of rice seedlings every 7 days for two weeks. The incidence of infection was the percentage of infected seedlings. 

### 2.3. Total RNA Extraction, RNA-Seq Library Construction, and Illumina Sequencing

Total RNA was extracted using an RNeasy Plant Mini Kit (QIAGEN, Germany) following the manufactures’ instructions. RNA concentration and purity were determined using NanoDrop 2000 (Thermo Fisher Scientific, Wilmington, DE). RNA integrity was analyzed using the RNA Nano 6000 Assay Kit of the Agilent Bioanalyzer 2100 system (Agilent Technologies, CA, USA).

Sequencing libraries were constructed using a NEBNext UltraTM RNA Library Prep Kit for Illumina (NEB, USA) following the manufacturer’s instructions. The cDNAs were generated using reverse transcriptase coupled with random primers, and adapters were ligated at both ends. The library fragments were purified with an AMPure XP system (Beckman Coulter, Beverly, USA). PCR was performed, and the products were assessed by an Agilent Bioanalyzer 2100 system. Then, the PCR products were sequenced on an Illumina platform (Nova-seq PE150), and paired-end reads were generated.

In order to obtain clean reads, the adaptors were removed from sequences, and reads containing poly-N and low-quality reads were removed. All clean reads were used for further analysis. The Hisat2 [18] was used to map the clean reads onto reference genome sequence. The reference genome used in this study is IRGSP 1.0. There are 35679 coding genes in the rice reference genome.

Gene expression levels were calculated by fragments per kilobase of transcript per million fragments mapped (FPKM). Differentially expression analysis was performed using DESeq2 [19] software. Genes with P-values lower than 0.01 were regarded as differentially expressed genes. The FDR (false discovery rate) < 0.01 and fold change ≥ 2 were set as the threshold of significantly differential expressed genes.

### 2.4. GO Enrichment Analysis and KEGG Enrichment Analysis

Blast2GO [20] software was used for Gene Ontology (GO) annotation. After GO annotation, the differentially expressed genes (DEGs) were divided into three categories: Molecular Functions, Biological Processes, and Cellular Component. KEGG analysis was generated using the Kyoto Encyclopedia of Genes and Genomes (KEGG) pathway online database [21]. 

### 2.5. Verification of Candidate Genes by Quantitative Real-Time PCR (qRT-PCR)

The expression level of transcripts putatively involved in phosphite-related genes was validated by qRT-PCR. The primers were designed using the NCBI online tool. First strand cDNAs were synthesized using SuperScriptTM III First-Strand Synthesis SuperMix (Invitrogen). SYBR1 Green PCR Master Mix (Applied Biosystems) was used, and the PCR products was quantified on a CFX384 Real-time PCR system (Bio-Rad). Three biological and three technical replicates were performed for each sample. The PCR profiles were as follows: 60 s at 95 °C for predenaturation, 40 cycles of 15 s at 95 °C for denaturation, and 25 s at 63 °C for annealing, followed by Melt-Curve analysis (55–95 °C, 0.5 °C increment for 5 s per step) to test the amplicon specificity. In order to normalize all the data, the amplification of Rice *eEF1α* sequence was used as an endogenous reference.

## 3. Results

**Figure 1:**
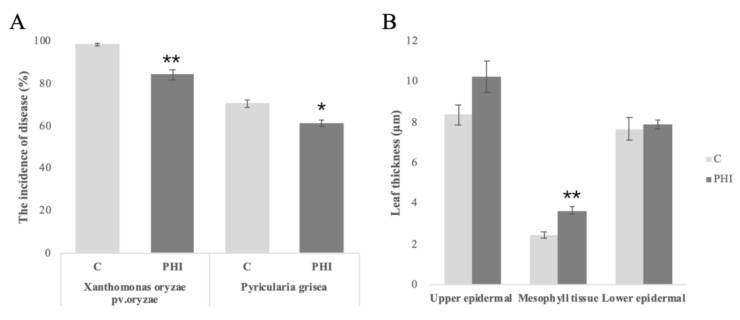
(**A**) The disease incidence rate of rice seedling between C (Control) and PHI (phosphite-treated) seedlings. (**B**) The thickness parameters of rice leaves between C (Control) and PHI (phosphite-treated) seedlings. Error bars represent the SE of three biological replicates. * *p* < 0.05; ** *p* < 0.01.

The incidences of *Xanthomonas oryzae pv.oryzae* and *Pyricularia grisea* were assessed after PHI application. Application of PHI resulted in a reduced susceptibility to *Xanthomonas oryzae pv.oryzae* and *Pyricularia grisea*. A reduction in the incidence of infection was observed. For these two pathogens, the incidence rate of *Xanthomonas oryzae pv.oryzae* and *Pyricularia grisea* was reduced by approximately 15% compared to control plants (Figure 1A). The leaf thickness parameters showed that leaves of PHI-treated seedlings were thicker both in epidermal and mesophyll tissues (Figure 1B).

To obtain an overview of the transcriptome profiling of the responsive genes to PHI in rice, RNA samples were prepared from the leaves of rice after PHI treatment. Gene expression profiles of the leaf samples were analyzed, and six cDNA libraries were constructed. After removing the low-quality reads, a total of 158,285,888 clean reads were generated. High quality sequences were mapped to the reference genome, 81–90% of the 24–31 million clean reads from each library were uniquely mapped reads, and around 8% were unmapped reads. Finally, we detected 22661 non-redundant genes in total, which were applied for further analysis. The RNA-sequencing data were deposited in the NCBI SRA database; the accession number is PRJNA664664.

**Figure 2:**
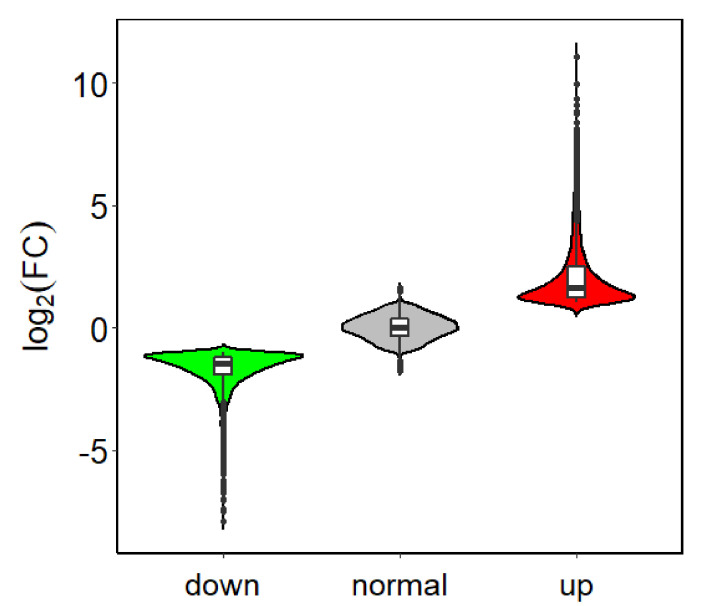
Violin plot of differentially expressed genes in C and PHI.

Using an FDR < 0.01and log2FC ≥ 1 as threshold values, 4311 genes were found to be differentially expressed between PHI-treated samples and the controls. Among them, 2064 genes (Table S1) were up-regulated and 2247 genes (Table S2) were down-regulated (Figure 2).

**Figure 3:**
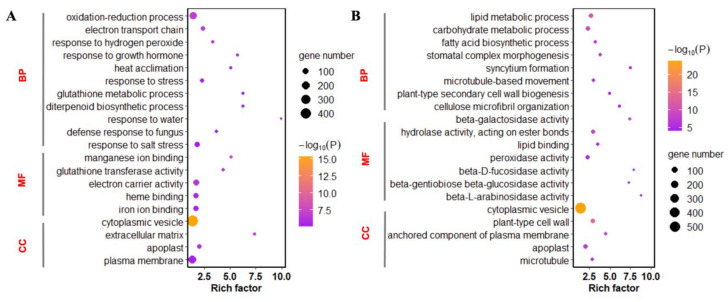
Gene Ontology (GO) enrichment analysis of up and down differentially expressed genes (DEGs) in C and phosphite (PHI). Up-regulated genes (**A**) and down-regulated genes (**B**).

The GO (Gene Ontology Consortium) annotation system contains three categories: biological processes (BP), molecular function (MF), and cellular components (CC). A total of 3572 genes of the differential expression genes (DEGs) were annotated by GO (Figure S1). The DEGs with known annotations could be categorized into 52 regulatory functional classes. The up-regulated genes were enriched in GO terms of response to stress, defense response to fungus, and plasma membrane. The GO enrichment of down-regulated genes refers to carbohydrate metabolic process, hydrolase activity, plant-type cell wall, and apoplast. The up-regulated genes were mainly enriched in defense-related processes, whereas down-regulated genes were enriched in metabolic processes (Figure 3).

**Figure 4:**
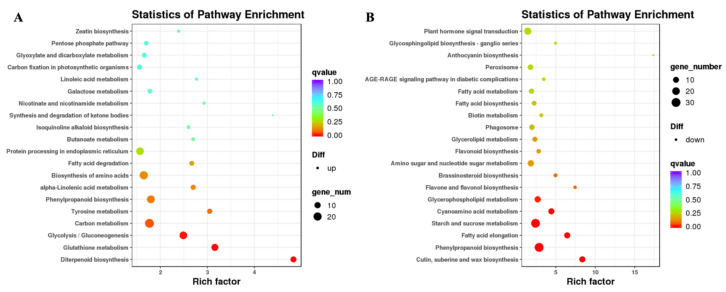
Kyoto Encyclopedia of Genes and Genomes (KEGG) pathway enrichment analysis based on the differentially accumulated genes in control and PHI-treated rice seedlings. Up-regulated genes (**A**) and down-regulated genes (**B**).

KEGG (http://www.genome.jp/kegg/) is a biological systems-related database, providing the mapping information of individual pathways. Among the DEGs annotated by KEGG pathway, 959 genes were mapped onto 114 KEGG pathways (Figure 4). These pathways included phenylpropanoid biosynthesis (ko00940), starch and sucrose metabolism (ko00500), and plant hormone signal transduction (ko04075). The PHI-induced DEGs were most enriched in phenylpropanoid and glycolysis pathways. There were 21 and 20 up-regulated genes, 34 and 6 down-regulated genes involved in phenylpropanoid and glycolysis pathways, respectively. Among the 25 DEGs identified in plant pathogen interaction pathway (ko04626), 14 genes were up-regulated, and 11 genes were down-regulated. There were 16 up-regulated and 20 down-regulated genes in plant hormone signal transduction.

**Figure 5:**
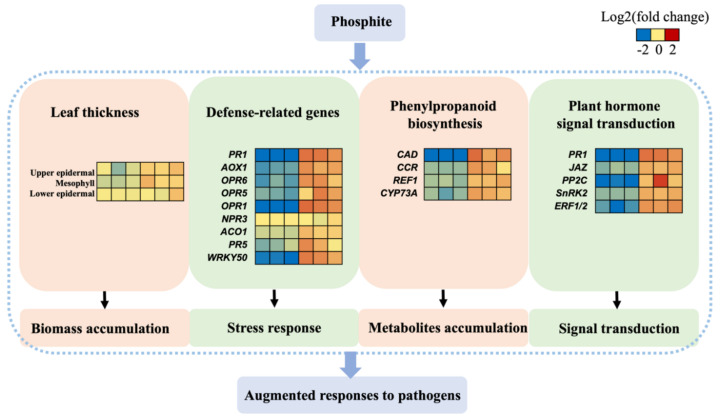
A predicted model of augmented responses of pathogens attack after PHI treatment. The intensity of orange and blue colors indicates the extent of induction and repression, respectively. For each heatmap, the first three columns are the controls and from fourth to sixth are the PHI-treated samples.

Genes related to phytohormone signaling pathways were analyzed. Thirty-six DEGs were identified in plant hormone signal transduction pathway. Some key genes in ethylene (*ERF1/2*), jasmonic acid (*JAZ*) and salicylic acid (*TGA* and *PR1*) pathway were up-regulated in PHI-treated seedlings (Figure 5). 

Transcription factors (TFs) are essential for the regulation of gene expression in plant development and growth. A total of 1623 transcripts encoding TFs were identified, in which 120 were up-regulated and 144 were down-regulated (Table S3). These TFs belonged to diverse families, including basic Helix-Loop-Helix proteins (bHLH), C_2_H_2_, Apetala 2/Ethylene-Responsive Element Binding Proteins (AP2/ERF-ERF), NAC Domain Containing Proteins (NAC), WRKY DNA-Binding Protein (WRKY), MYB Domain Proteins (MYB), and basic region/leucine zipper motif (bZIP). 

Protein kinases are key regulators which contains diversified gene families. They play roles in the transfer of phosphate groups to substrate proteins. PHI induced 225 DEGs encoding kinases including receptor-like protein kinase (RLK), calcium-dependent protein kinase (CAMK), and mitogen-activated protein kinases (MAPKs).

The expression levels of genes related to PHI treatment were verified by qRT-PCR (Figure S2). The expressions of *ACO1*, *OPR1*, and *PR1* under PHI treatment were significantly higher than controls. The expression pattern obtained from qRT-PCR was consistent with RNA-sequencing result.

As shown in the heatmap (Figure 5), most of the defense genes were up-regulated in response to PHI treatment. The expression of pathogenesis-related protein 1 (PR1) showed the largest fold change of up-regelation in PHI-treated seedlings compared with controls. It was up-regulated by 16.76-fold. *NPR3* and *ACO1* had similar expression levels both in PHI treatments and the controls. PHI increased transcript levels of the rest of the defense-related genes.

## 4. Discussion

PHI is extensively applied to plants for the protection of diseases mainly caused by oomycetes [22]. In this study, we investigated the effect of PHI (PHI) on the transcriptome profile in rice seedlings. Our results showed that treatment of rice seedlings of PHI conferred tolerance against *Xanthomonas oryzae pv.oryzae* and *Pyricularia grisea*.

The effect of PHI on disease control was studied using *Xanthomonas oryzae pv.oryzae* and *Pyricularia grisea*-incubated rice plants. The application of PHI resulted in a significantly reduced disease incidence after inoculation of *Xanthomonas oryzae pv.oryzae* and *Pyricularia grisea*. The durability and the systemic defense of a broad range of pathogens were achieved with the application of PHI. It is an effective fungicide in controlling *Xanthomonas oryzae pv.oryzae* and *Pyricularia grisea* incidence rate in rice. Protection effects of different PHI compounds against *P. infestans* were observed in foliage and in post-harvest tubers [23].

Compared with traditional fungicides, PHI was both effective and eco-friendly. For example, PHI exhibits an equal effect against *Penicillium expansum* in apples compared with thiabendazole [24]. Priming phenomena were induced when PHI concentration was 10 mM or less, and PHI inhibited the growth of pathogens directly at higher doses [17]. Lobato et al. (2011) reported that the tuber weight was higher in 1% KPHI-treated potatoes than the controls. The defense against disease and the yield were both increased in potatoes after foliar treatment of KPHI [9]. Less Phytophthora crown rot incidence was observed in PHI-treated pepper plants compared with controls and phosphate-treated plants [12]. This is consistent with a study verifying the antifungal effect of PHI against oomycetes [25].

PHI has been used as a fungicide as it could prime defenses for augmented responses to pathogens [16]. The results obtained from this study confirmed that PHI induces the resistance against pathogen attack via priming. PHI application reduced the incidence of *Xanthomonas oryzae pv.oryzae* and *Pyricularia grisea*. The main aim of this study was to discover the mechanism of defense effect of PHI against pathogens. In this study, we investigated the effect of PHI application on resistance against pathogens at the transcriptional level. We analyzed the expression of defense genes, which were involved in SA or JA/ET pathways. It was concluded that the PHI-induced up-regulation of defense genes could be one of the explanations of priming phenomena. The induced thickness of epidermal and mesophyll tissues was consistent with the accumulation of callose, which was regarded as one of the PHI-induced priming strategy [26,27].

SA is an essential element required in plant defense signaling pathways. In susceptible varieties of potatoes, the gene expression of PRs and SA was significantly lower than potatoes, which showed higher resistance to pathogens. JA and ET pathways were also involved in priming responses. Some key genes in ethylene (*ERF1/2*), jasmonic acid (*JAZ*), and salicylic acid (*TGA* and *PR1*) pathways had higher expression levels in PHI-treated seedlings [28]. *ACO1* was related to the JA/ET pathway [11,29]. The expression level of *ACO1* was higher in PHI-treated seedlings than controls. This was consistent with the finding revealed by RNA-seq.

The expression of PR1 was promoted under PHI treatment. *OPR1* belongs to the defense-related pathway [29]. *PR1* and *OPR1* expressions were significantly induced by PHI treatment. WRKY transcription factors are important in the regulation of SA-dependent defense responses. The expressions WRKY transcription factors were up-regulated in PHI-treated seedlings. 

The expression levels of genes in the phenylpropanoid pathway were also induced in PHI treatments (Figure 5). In the phenylpropanoid pathway, many phenolics were synthesized. Phenolics were induced during the process of pathogen attack, and it was important in the defense response. Transcripts involved in the phenolic synthesis pathway were significantly changed after PHI treatments. Araujo et al. (2015) found that mango plants sprayed with PHI reduced the Ceratocystis fimbriata development. The concentrations of phenolic compounds were higher in mango plants sprayed with PHI [8]. 

A transcriptome profiling was conducted to clarify the molecular mechanisms underlying PHI-induced resistance to pathogens. As reported earlier, the priming phenomenon could induce a series of biological responses, such as the reinforcement of the cell wall, the up-regulation of pathogen-related genes, and the accumulation of ROS. The ability of defense against pathogens was primed after PHI incubation. Taken together, our results supported the conclusion that PHI treatment could boost the resistance of rice seedlings against some pathogen attacks (Figure 5). Genes related to hormone signaling and defense responses were augmented under PHI treatment which induced defense responses. The DEGs discovered in this study could provide a step towards understanding the molecular mechanism of priming phenomena. The induced defense genes could be utilized in rice breeding of superior resistant cultivars or novel fungicides in the future.

## Supplementary Materials

The following are available online at https://www.mdpi.com/2223-7747/9/10/1334/s1. Table S1: Annotations and expression values of the up-regulated differentially expressed genes (DEGs). Table S2: Annotations and expression values of the down-regulated differentially expressed genes (DEGs). Table S3: List of differentially expressed transcription factors. Table S4: The primers used in the qRT-PCR. Figure S1: GO classification of DEGs in C and PHI. Figure S2: qRT-PCR analysis of genes related to PHI treatment.
