# Supplementary figures and images for: Transcriptome-Based Analysis of Phosphite-Induced Resistance against Pathogens in Rice
Source: Plants (Basel). 2020 Oct 9;9(10):1334. doi: 10.3390/plants9101334 (PMC7650589; doi:10.3390/plants9101334)

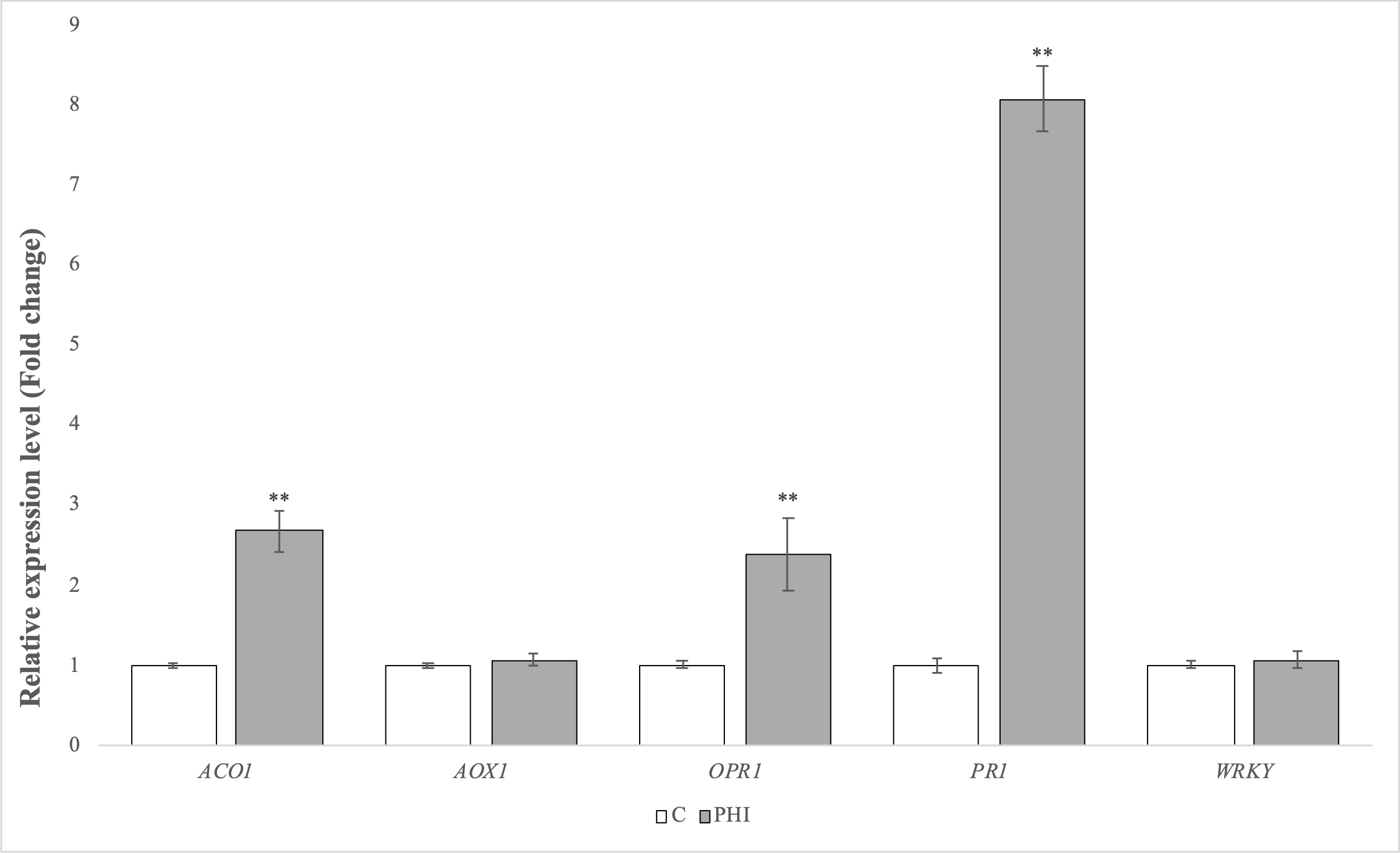

Supplement: Supplementary file 1 [file plants-09-01334-s001.zip › supplementary files/Figure S2.tiff]
